# Effects of Sex and Reproductive State on Interactions between Free-Roaming Domestic Dogs

**DOI:** 10.1371/journal.pone.0116053

**Published:** 2014-12-26

**Authors:** Jessica Sparkes, Gerhard Körtner, Guy Ballard, Peter J. S. Fleming, Wendy Y. Brown

**Affiliations:** 1 School of Environmental and Rural Sciences, University of New England, Armidale, Australia; 2 Vertebrate Pest Research Unit, Biosecurity New South Wales, Armidale, Australia; 3 Vertebrate Pest Research Unit, Biosecurity New South Wales, Orange, Australia; 4 Invasive Animals Cooperative Research Centre, Armidale, Australia; University of Sydney, Australia

## Abstract

Free-roaming dogs (*Canis familiaris*) are common worldwide, often maintaining diseases of domestic pets and wildlife. Management of these dogs is difficult and often involves capture, treatment, neutering and release. Information on the effects of sex and reproductive state on intraspecific contacts and disease transmission is currently lacking, but is vital to improving strategic management of their populations. We assessed the effects of sex and reproductive state on short-term activity patterns and contact rates of free-roaming dogs living in an Australian Indigenous community. Population, social group sizes and rates of contact were estimated from structured observations along walked transects. Simultaneously, GPS telemetry collars were used to track dogs' movements and to quantify the frequency of contacts between individual animals. We estimated that the community's dog population was 326±52, with only 9.8±2.5% confined to a house yard. Short-term activity ranges of dogs varied from 9.2 to 133.7 ha, with males ranging over significantly larger areas than females. Contacts between two or more dogs occurred frequently, with entire females and neutered males accumulating significantly more contacts than spayed females or entire males. This indicates that sex and reproductive status are potentially important to epidemiology, but the effect of these differential contact rates on disease transmission requires further investigation. The observed combination of unrestrained dogs and high contact rates suggest that contagious disease would likely spread rapidly through the population. Pro-active management of dog populations and targeted education programs could help reduce the risks associated with disease spread.

## Introduction

Free-roaming dogs (*Canis familiaris*) occur in many parts of the world [1], displaying a wide diversity of population sizes and social organisations, ranging from solitary individuals to members of large social groups [2]–[4]. The variation in sociality of these canids is often in response to population size and resource availability, including food, shelter and potential mates within an area [5]–[8]. Management programs often involve capture, veterinary treatment and neutering, with the objective of reducing population growth and improving the overall health of the free-roaming dog population, associated humans, other domestic pets and wildlife (e.g. [9], [10]). However, information on the effect of sex and reproductive state (and hence neutering) on contact rates and movements is limited and appears to be location and context specific [11]–[13].

In Australia, free-roaming dogs are commonly associated with remote communities where dogs live and search for food around dwellings and community infrastructure [14], [15]. In some remote Indigenous communities, dogs have cultural, spiritual and physical significance, which must be accounted for in dog management programs [16], [17]. However, these dogs also spread parasites and disease, such as sarcoptic mange (*Sarcoptes scabiei*), hookworm (*Ancylostoma caninum*), *Giardia duodenalis*, heartworm (*Dirofilaria immitis*), ticks (*Rhipicephalus sanguineus*) and fleas [18] to humans and wildlife [19], [20]. Neutering and veterinary care programs to limit dog population growth and reduce the incidence of both dog and human disease are underway in many of these communities [21].

Despite the risk that these dogs pose to humans and other animals, population level studies are sparse [19], [22]. In particular, key knowledge of the effects that sex and neutering programs have on movement behaviour and rates of contact between individual dogs is lacking. Understanding these factors is essential for determining the extent, appropriate timing and duration of dog health programs in these communities, while also providing essential parameters for endemic and epizootic disease modelling [16], [23], [24].

Here, we quantified the dog population and social unit size, activity patterns and contact rates, and determined whether sex and neutering affected these parameters within a free-roaming dog population associated with an Aboriginal island community in northern Australia.

## Method

### Study site

The study was conducted in the Wurrumiyanga community (11.76°S, 130.64°E) on the south-east corner of Bathurst Island, within the Tiwi Islands group, Northern Territory, Australia. Bathurst Island spans an area of 169 300 ha, is tropical and mostly covered by tall eucalypt forests, interspersed with rainforest patches and mangroves. The average annual rainfall is 2 035mm; January is the wettest month and July the driest. Average maximum daytime temperature is 31.2°C for the dry season, when the study was conducted (June/July) and 33.4°C immediately prior to the wet season (Oct/Nov) [25]. Mean minimum temperature for the same periods are 18.6°C and 23.7°C, respectively [25].

### Population estimation

Over five consecutive days, sightings of all dogs, including known, collared and otherwise marked dogs were recorded by a team of two observers moving on foot along a pre-defined 5.5 km transect, covering 58% of the town's roadways, at 0700, 1200 and 1700 hrs (*N* = 15 transects). To aid identification, observers used a digital video camera to record all dogs observed on the transect. The number of dogs sighted, their confinement status and group sizes were also recorded. The Chapman [26] estimator, which is unbiased for small recapture samples, was used to estimate the total dog population from repeated sightings of known marked dogs for each transect walk and the mean calculated from the 15 estimates.

### Study animals for GPS tracking

Twenty dogs (2 entire females, 7 spayed females, 6 entire males and 5 neutered males) were recruited for Global Positioning Satellite (GPS) tracking by opportunistically asking community members to volunteer their pets. All study dogs were owned by residents but were unrestrained and allowed to roam freely about the community, which is normal for these animals. To record their movements and contacts, each dog was fitted with a Mobile Action (Taiwan) i-gotU GT-120 low-cost GPS-tracking device, mounted on an off-the-shelf dog collar (total weight 230 g). GPS-devices were programmed to record locations at 15 minute intervals. Data were downloaded upon retrieval of collars from the dogs.

The relatively short deployment period of the GPS collars (7 days) was deemed sufficient to provide areal context for observations along transect walks, but precluded calculation of traditional home ranges [27]. Therefore, the area encompassing the GPS fixes was termed an ‘activity range’ (AR), and pertained to the observational period only. The AR of each collared dog was calculated as a Minimum Convex Polygon (MCP; [28]). Based on the location data, the accumulated distance (adding all distances between each pair of successive data points) was calculated each day. Sex and reproductive effects for AR and accumulated distance were determined by running a 2-way ANOVA using R statistical software version 3.0.2 [29].

Contacts between pairs of collared dogs were identified by searching through all GPS data sets for concurrent location fixes (±7.5 min) that were less than 20 m apart. Twenty meters was chosen as the contact threshold based on the effective accuracy of the GPS units. This decision was supported by observations along walked transects indicating that physical or close contact was likely to be elicited when two or more individuals sighted each other at this distance or shorter. The duration of contacts (an ‘event’) was calculated as the length of an uninterrupted string of contact records (i.e. number of contact records multiplied by 15 min). Subsequently, the contact data were searched for concurrent events involving two or more collared dogs to determine group size during a contact event. A 2-way ANOVA of dog sex (male, female) and reproductive state (neutered, entire) effects on contacts was conducted.

To determine if the number of recorded contacts for a pair of dogs were likely to be a result of random encounters of dogs moving within their AR, we simulated random encounters by using the original time and distance between subsequent location records but randomised direction of movement, constrained within the real AR for each dog. The average of 50 simulations was used to represent the number of chance encounters between a pair of dogs. As the AR of some dogs did not overlap, pairs with zero random encounters were removed from further analyses. Actual contacts for the remaining pairs were compared with simulated contacts and evaluated using a Welch's two sample *t*-test [30]. The whole procedure (both actual and simulated contacts), as well as the daily distance calculations and AR estimates were performed with programs written in Visual Basic 6.0 (Microsoft Corp.) by one of the authors (G.K). The programs enable the user to set parameter limitations that prevail in the dataset analysed (e.g. duration between successive fixes, altering distance threshold), ensuring application to a wide variety of datasets. Data are presented as mean ±1 standard deviation; *N* =  number of observations.

Research was conducted with approval from the UNE Animal Ethics Committee (AEC13-009). Fieldwork was carried out with permission from Tiwi Islands Shire Council.

## Results

During transect walks, an average of 147±18 dogs were observed, with only 9.8±2.5% of dogs confined to a yard. Including the 20 GPS-collared dogs, 163 dogs were individually recognisable and all of these dogs were resighted during the transect walks. Subsequently, the community dog population was estimated at 326±52 (*N* = 15), with an average of 1.1 dogs per household (21 dogs per 100 people; data based on [31]). This represents all dogs within the community, including owned free-roaming, confined and stray dogs greater than 6 weeks of age.

**Figure 1:**
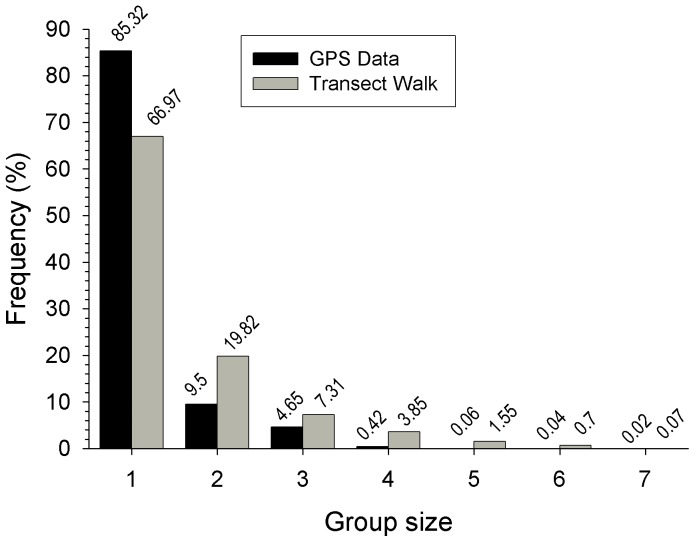
Comparison of social group sizes between GPS-collar and transect data.

Consequently, the sample of dogs fitted with GPS collars (*N* = 20) represented 6.1% of the population. Data were retrieved from 17 dogs (i.e. 5.2% of dog population); two collars were lost, while another became dysfunctional when bitten by a dog shortly after deployment. Solitary dogs were observed most often, with social groups ranging in size from 2 to 7 individuals. Size of social groups, determined from transect observations (*N* = 1 423 groups observed), correlated strongly with those estimated for the GPS-collared dogs (transect walk  =  GPS data*0.76+3.48, *r^2^* = 0.97, P<0.001; Fig. 1). Overall, GPS-collared dog social groupings were smaller compared with transect observations because not all dogs were collared within the community.

Overall, a total of 7 165 GPS fixes were recorded, with a GPS fix success rate of 73±14.4%. By chance, one GPS collar remained functional on the dog (#16) for 22 days, well beyond the calculated battery life of 7 days. This data set was used to compare its AR data with other shorter deployment durations. During the monitoring period, one dog undertook a long distance foray into the surrounding bush (17 km). This foray was regarded as exploratory (*sensu* [32]), falling outside the normal AR of the animal and was thus excluded from its AR and movement analyses.

**Figure 2:**
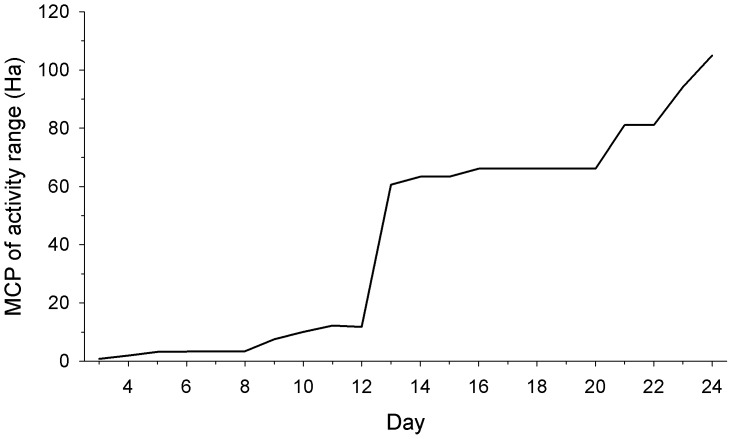
Cumulative Activity Range of Dog 16 (Minimum Convex Polygon in 1 day increments) demonstrating abrupt changes in activity range.

The AR for collared dogs over the 7 days of deployment varied from 9.2 to 133.7 ha (mean AR = 51.0±36.1; Table 1) and the mean daily distance travelled was 3 169±980 m (Table 1). The cumulative AR for Dog 16 over 21 days showed two marked plateaus (Fig. 2), corresponding to a shift in its core area during the monitoring period. Despite this, the AR of this dog fell within the limits of other neutered male dogs with the shorter GPS deployment, indicating that short-term ARs were relatively stable and provided an appropriate timeframe for measuring contact rates.

**Table 1:** Activity range and distance travelled per day for free-ranging community dogs, northern Australia.

Dog ID	Sex#	Activity Range (ha)	Distance (m/day)
9	FE	30.75	3 193
12	FE	68.6	3 798
1	FS	9.16	1 591
2	FS		
5	FS	37.47	3 514
14	FS	40.81	2 445
17	FS	18.12	2 012
18	FS	29.92	2 030
19	FS	18.56	2 399
4	ME	25.4	4 031
6	ME	14.97	2 364
7	ME	89.54	3 748
8	ME	76.5	4 168
15	ME		
20	ME	33.83	2 774
3	MN	88.34	4 446
10	MN	46.09	3 243
11	MN	133.69	5 182
		(2293.09∧)	
13	MN		
16	MN	105.01	2 935

# FE: Female Entire; FS: Female Spayed; ME: Male Entire; MN: Male Neutered.

∧ Value incorporates foray undertaken during the study period.

A 2-way ANOVA yielded a significant main effect for sex; male collared dogs utilised a larger area than females (mean AR = 68.15±40.17, N = 9; 31.67±18.31, N = 8, respectively), while there was no effect of reproductive state on AR (Table 2). However, the interaction effect was significant; indicating that neutered male dogs had significantly larger ARs than spayed female dogs (mean AR = 93.28±36.61, N = 4; 25.67±12.38, N = 6, respectively). Similarly, a significant main effect showed that overall, males travelled further each day compared with females (mean distance travelled  = 3 654±901, N = 9; 2 623±790, N = 8, respectively, Table 2).

**Table 2:** Effect of sex (male, female) and reproductive state (entire, neutered) on activity range, accumulated distance moved and contacts (*N* = 17 dogs).

	F_1_ value	Pr (>F)
**Activity Range**		
Sex effect	7.442	0.02
Reproductive state	1.477	0.25
Sex effect*Reproductive state	5.668	0.03
**Accumulated Distance**		
Sex effect	7.137	0.02
Reproductive state	0.132	0.72
Sex effect*Reproductive state	4.088	0.06
**Contacts**		
Sex effect	1.201	0.29
Reproductive state	0.060	0.81
Sex effect*Reproductive state	7.191	0.02
**Number individuals contacted**		
Sex effect	0.270	0.6
Reproductive state	0.001	0.98
Sex effect*Reproductive state	5.639	0.03

**Figure 3:**
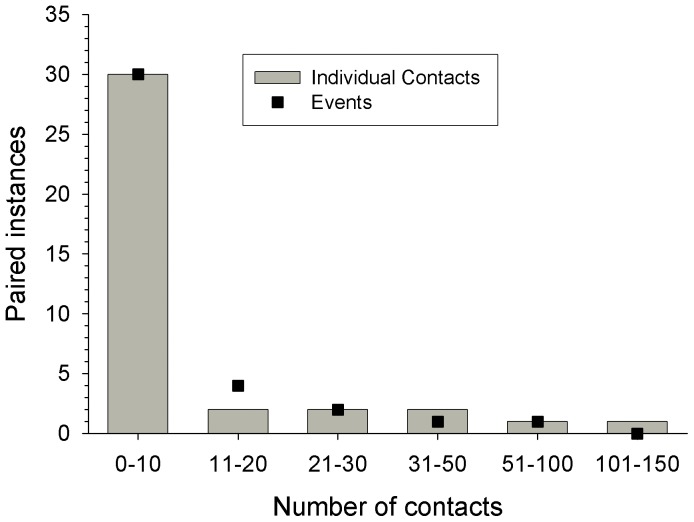
Number of individual contacts (columns) and proximity events (points) between paired GPS-collared dogs ≤20 m apart.

A total of 412 contacts, from 271 separate proximity events were recorded between pairs of collared dogs (mean: 5.24±5.30 contacts per dog per day; Fig. 3). During the 7 days, 4 789 GPS fixes (85%) did not result in contact with another collared dog (i.e. a ‘solitary’ dog; Fig. 1).

Depending on sex, neutering had the opposite effect on contacts, where neutered male and entire female dogs (mean contacts  = 14.13±10.97, *N* = 4; 16.17±10.84, *N* = 2, respectively) had significantly more contacts with other collared dogs compared to spayed females and entire males (mean contacts  = 2.35±3.09, *N* = 6; 6.43±7.46, *N* = 5, respectively, Table 2). There was no observable main effect of sex or reproductive state on contacts during the study period (Table 2). Event duration ranged from 15 minutes to 2.5 hours, with most events lasting 15 minutes (i.e. a single contact record).

Observed contact rates differed significantly from simulated contact rates for collared dogs (Welch *t* test: *t*_113_ = 2.55, *P* = 0.01). “Avoidance behaviour” (i.e. where the number of observed records was significantly less than those predicted from random encounters; <95% confidence interval) was recorded on 48 paired-collar instances, with the highest “avoidance” observed for dogs 07 and 17 (0 vs 2.24, 1 vs 3.14; actual vs simulated, respectively). In contrast, the number of recorded contacts was higher than the 95% confidence limit of the simulation for 37 pairs of dogs, while 30 pairs of collared dogs matched the simulation prediction.

Group size of collared dogs ranged from 1 to 7 individuals, with most contacts occurring between two dogs only (Fig. 1). The number of different collared dogs that an individual collared dog encountered during the study period ranged from 0 to 10 (mean individuals encountered  = 4±2.8, *N* = 17). Similar to contact rates, there was no effect of sex or reproductive state on the number of dogs an individual came into contact with (Table 2). However, there was a significant interaction effect, where neutered male and female entire dogs (mean individuals contacted: 6.3±2.9, *N* = 4; 7±1.4, *N* = 2, respectively) contacted more dogs compared with male entire and female spayed dogs (mean individuals contacted: 3.6±3.4, *N* = 5; 3.2±1.7, *N* = 6; *P* = 0.03, respectively; Table 2).

## Discussion

Short-term activity ranges of the monitored free-roaming domestic dogs on Tiwi Islands were small relative to the reported home ranges of dingoes and other wild dogs from semi-arid and arid Australia [3], [11], [33]. Nevertheless, males still had significantly larger ARs than females, which is consistent with the pattern seen in longer studies of wild dogs (e.g. [3], [11]). Contacts between individually collared dogs were frequent, but on average, entire female and neutered male dogs contacted significantly more dogs than spayed females and entire males respectively.

During the study period, dog ownership within the community was estimated at 1.1 dogs per household (21 dogs per 100 people), which is consistent with global estimates (1.1; [20]) and only slightly higher than the Australia-wide estimate of 16 dogs per 100 people [34]. More importantly, very few dogs were confined. Community members' attitudes towards dog ownership and containment typically mean that their dogs are allowed to roam freely like any other member of the family [21]. Consequently, the probability of contacts occurring between dogs was high and groups of up to 7 individuals were observed. Similar to Rubin and Beck [35], contact events (i.e. social groupings) were usually transitory aggregates, with the majority of events lasting less than 15 minutes. These transitory social groupings increase the probability of contacts occurring between a greater number of individuals, potentially increasing the rate of disease transmission.

However, as ARs of the study dogs were generally confined to the community boundaries, this would likely limit disease transmission to the community confines in the first instance. One dog did however, undertake a long distance foray towards the local rubbish tip. Interestingly, this dog was frequently observed within the town boundaries with its litter mate (#10) during transect walks, but the latter dog did not undertake a similar foray while collared. This was reflected in the high number of contacts between the pair but varying ARs and daily accumulated distances travelled even with the foray excluded (Table 1); with Dog 11 travelling further each day and over a larger area, suggesting the tendency of this dog to roam widely.

In a global context, Vaniscotte et al. [12] estimated similar ARs to our study for free-roaming domestic dogs in Tibet (range 32.5–174.5 ha, *N* = 78), while other studies have estimated much smaller home ranges, ranging from 2 to 10 ha for free-roaming domestic dogs in Australia, Indonesia and the USA [22], [35]–[38]. Meek [19] estimated much larger home ranges for free-roaming dogs from an Australian Indigenous community in coastal south-east New South Wales (range: 140–2 450 ha, *N* = 10). Those dogs conducted regular forays into the surrounding bush and hence resemble the Tiwi Island Dog 11 estimate when including its foray (AR = 2 293). The differences in ARs, home ranges and the propensity to undertake forays between studies may relate to methodological differences (e.g. duration of the monitoring period) and the size of the communities where studies were conducted. Resource availability; including food, shelter, companionship and barriers (natural or manmade) may have also affected effective ARs. Dogs living in resource rich areas tend to have smaller home ranges compared with resource poor areas, which reduce the need for the animal to travel greater distances to meet its biological and social requirements [2], [3], [8], [19].

Some of the variability within the dog population was evidently related to sex and reproductive status. Collectively, male dogs travelled further each day and over a larger area than females. These findings are in agreement with Thomson [11] and Vaniscotte et al. [12], but in contrast to Van Kesteren et al. [13] and Dürr and Ward [22], where no difference between male and female AR or distance traveled per day was found. At any rate, the larger area covered by males in the present study may increase their potential to spread diseases further through the community than females. For diseases that require physical contact (e.g. rabies, ringworm); contact between susceptible individuals would have greater significance for disease transmission. In the case of Tiwi Islands, an average of 5.24 contacts per collared dog per day was recorded and if extrapolated to the entire dog population, a contact rate of 101 per dog per day would be expected.

There were large variations in contacts for collared individuals, ranging from 0 to 24 contacts per day. Individual circumstances were important and some of the very high contact rates could be explained by cohabitation. For example, Dogs 10 and 11 were owned by the same household and recorded 105 contacts between the pair. In contrast, Dogs 08 and 12 came from a single household, but only recorded 42 contacts between the pair. As these dogs were not confined, individual dogs were allowed to express avoidance behaviour, even within a cohabitation environment.

In addition to obvious individual characteristics observed within the data, global trends for sociality were also evident. In contrast to AR and distance travelled, there was no significant difference in the number of contacts recorded between collared male and female dogs. However, there was a significant effect of reproductive state on contacts, with entire females and neutered males contacting more dogs than spayed females and entire males. Neutering has been found to disrupt sociality of dogs, removing the dominance hierarchy [39], while also increasing activity in domestic dogs [40], creating more opportunities for contact with other dogs. Multiple-mate matings [5], [39] and interactions with neutered dogs (i.e. lack of perceived competition for resources), may have resulted in the higher contacts recorded for entire females. As there is no clear breeding season on the Tiwi Islands; with dogs able to breed any time of year (S. Cutter, Pers. Comm.), confinement strategies tailored to the individual would need to be implemented to reduce these contacts. However, only two entire females were collared during the present study and further research is required to determine whether this effect is consistent at a population level. In contrast, dominance and territorial aggression was found to be more common in male dogs [41], [42], which may result in fewer contacts due to avoidance behaviour exhibited by subordinate animals.

Our data describes short-term movements and provides the first quantitative assessment of contacts between free-roaming domestic dogs in northern Australia. This information is a critical precursor for modelling endemic and exotic diseases of community dogs, and devising appropriate control programs. Observed high contact rates, fidelity to home and the transitory nature of dog social groupings combined with a lack of confinement indicates the potential of a disease to spread rapidly through this and similar communities, with limited/delayed spread to the surrounding landscape. Spayed females did have significantly fewer contacts than entire females, suggesting that neutering programs targeted towards the female portion of the population would be beneficial in reducing contact rates and hence, opportunities for breeding as well as reducing the predicted rate of spread of contagious diseases.
